# Traditional Birth Attendant reorientation and Motherpacks incentive’s effect on health facility delivery uptake in Narok County, Kenya: An impact analysis

**DOI:** 10.1186/s12884-017-1307-7

**Published:** 2017-04-21

**Authors:** John Emmanuel Kitui, Vaughan Dutton, Dirk Bester, Rachel Ndirangu, Susan Wangai, Stephen Ngugi

**Affiliations:** 1 Christian Aid, P.O. Box 13864, Westlands, 00800 Nairobi Kenya; 2 St Cross College, St Giles, Oxford, OX13LZ UK; 3 Wolfson College, Linton Road, Oxford, OX26UD United Kingdom

**Keywords:** Traditional Birth Attendants, Skilled delivery, Maternal mortality, Non-financial Incentives, Maternal and child health, Community health workers

## Abstract

**Background:**

A community health programme in Narok County in Kenya aimed to improve skilled birth assistance during childbirth through two demand side interventions. First, traditional birth attendants (TBAs) were co-opted into using their influence to promote use of skilled birth attendants (SBAs) at health facilities during delivery, and to accompany pregnant women to health facilities in return for a Ksh500 (Approximately USD5 as of August 2016) cash incentive for each pregnant mother they accompanied. Secondly, a free Motherpack consisting of a range of baby care items was given to each mother after delivering at a health facility. This paper estimates the impact of these two interventions on trends of facility deliveries over a 36-month period here.

**Methods:**

Dependency or inferred causality was estimated between reorientation of TBAs and provision of Motherpacks with changes in facility delivery numbers. The outcome variable consists of monthly facility delivery data from 28 health facilities starting from January 2013 to December 2015 obtained from the District Health Information Systems 2 (DHIS2). Data were collected on the 13th, 14th or 15th of each month, resulting in a total of 35 collections, over 35 months. The intervention data consisted of the starting month for each of the two interventions at each of the 28 facilities. A negative binomial generalized linear model framework is applied to model the relationship as all variables were measured as count data and were overdispersed. All analyses were conducted using R software.

**Findings:**

During the 35 months considered, a total of 9095 health facility deliveries took place, a total of 408 TBAs were reached, and 2181 Motherpacks were distributed. The reorientation of TBAs was significant (*p* = 0.009), as was the provision of Motherpacks (*p* = .0001). The number of months that passed since the start of the intervention was also found to be significant (*p* = 0.033). The introduction of Motherpacks had the greatest effect on the outcome (0.2), followed by TBA intervention (0.15). Months since study start had a much lower effect (0.05).

**Conclusion:**

Collaborating with TBAs and offering basic commodities important to mothers and babies (Motherpacks) immediately after delivery at health facilities, can improve the uptake of health facility delivery services in poor rural communities that maintain a strong bias for TBA assisted home delivery.

**Electronic supplementary material:**

The online version of this article (doi:10.1186/s12884-017-1307-7) contains supplementary material, which is available to authorized users.

## Background

Skilled help during child delivery is an important part of improving maternal health outcomes, particularly in developing countries where maternal mortality is high [1]. This is especially the case because most maternal deaths result from causes that are preventable or effectively manageable with the correct expertise, such as postpartum bleeding, complications from unsafe abortion, hypertensive disorders, postpartum infections and obstructed labour [1–3]. The provision of “accredited health professional[s], such as a midwife, doctor or nurse…educated and trained to proficiency in the skills needed to manage normal (uncomplicated) pregnancies, childbirth, and the immediate postnatal period”, can vastly reduce birth-related mortality” [2]. If, in addition to this, the health professional is trained in the “identification, management and referral of complications in women and newborns”, and is backed up by emergency referral services to specialised facilities in the event of complications happening in rural facilities, maternal deaths can be reduced even further [4, 5]. Access to skilled birth attendants (SBAs) in health facilities able to offer them a context in which to work effectively can have a significant impact on maternal and child health outcomes.

Although many low and middle income countries have managed to implement measures to this effect, overall uptake of SBAs during childbirth has been low. Approximately 39% of Kenyan women still deliver away from skilled help offered at health facilities, often choosing to deliver under the care of Traditional Birth Attendants (TBAs) instead of attending health facilities [6].^1^ As a result, TBAs have often been negatively portrayed as an obstacle to the reduction of childbirth mortality rates. A more positive view, however, frames TBAs as presenting a means whereby women delivering away from accredited health professionals could be accessed, and through which mortality reducing measures might be implemented. This logic was in place since the 1970’s, during which time efforts were made to train TBAs to identify life threatening complications early and to refer these to specialised care. These measures failed to significantly improve uptake of skilled deliveries and to improve maternal health indicators. Unfortunately, by the end of the 1990s, reviews began to provide evidence that these measures had been either ineffective or evidence inconclusive on their overall impact in reducing childbirth mortality [7], and by the turn of the century it had become generally accepted that TBAs were an unacceptable substitute for skilled assistance at birth, for all cases of childbirth [8, 9]. The strategy for improving maternal and neonatal health outcomes then shifted toward suppressing TBA activity in favour of all childbirth taking place in health facilities in the presence of SBAs. Low and middle income countries (LMICs) implemented a wide range of interventions in an effort to enact this strategy, including, amongst others, free at-the-point-of-delivery services, criminalisation of TBA services, cash transfers, vouchers and equity funds [9–11]. Alongside this, most safe motherhood programmes took measures to co-opt TBAs by retraining them to promote health facility based delivery [12].

Reviews and evaluations of these interventions typically yielded disappointing or unclear results. When taking maternal and new-born mortality rates as the outcome variable, there appeared to be little or no effect; although a survey by the Kenyan government, taking the same outcome, found partial success; perinatal and neonatal deaths were reduced, but not maternal mortality. [6]. Other assessments took the rate, detection, and referral of postpartum complications as the outcome, but the evidence was found to be inconclusive for overall increases in the detection of complications, in referral to the formal health care system, and in the utilization of essential obstetric services among mothers [13].

The above studies did not assess the impact of reorienting TBAs from conducting deliveries themselves to promoting SBA assisted deliveries at health facilities. Other studies have done so, and suggest that reorientation can lead to increased uptake in situations where TBAs are well integrated within the health system, and are offered an enabling environment in which to fulfil their new role. An examination of such an intervention in Hargeisa, Somaliland, found a 200% increase in the number of facility-based deliveries from 993 to 2872 per year [14]. This suggested a positive interaction between TBA reorientation and enabling environment (in this case, additional facility support and a strengthened referral system) when the outcome is maternal and newborn mortality. Similarly, positive findings were forthcoming in Tanzania and Uganda, where training of TBAs in the identification of high risk pregnancies was found to relate to an increase in the uptake of facility level obstetric health care [15, 16]. A systematic review of the effect of linking TBAs with formal health workers on the outcomes of SBA frequency, referrals, and facility deliveries, showed a positive effect. This provided additional evidence that the integration of TBAs with formal health systems can increase SBA deliveries and thereby impact on childbirth mortality [17]. The greatest impact was seen when interventions sought to overcome context-specific barriers to contact between SBAs, TBAs, and women.

Demand-side financial incentives such as cash transfers and vouchers have been found to be effective mechanisms in increasing uptake of public health services among mothers in resource-poor contexts, but did not necessarily address non-financial barriers [9, 17]. We did not find any study that has evaluated the use of non-financial incentives such as mother-packs to increase facility deliveries.

In 2013, a maternal and child health (MCH) programme was initiated in Narok County in Kenya with the intention of improving maternal and neonatal health outcomes through increasing the uptake of skilled delivery at health facilities [18]. Two new interventions were introduced by this programme: traditional birth attendants (TBAs) were to be incorporated into the formal health systems by reorienting them through education and a cash incentive of Ksh500^2^ per woman referred. The objective of this reorientation was to encourage them to promote health facility deliveries amongst pregnant women, and to encourage TBAs to accompany mothers in labour to health facilities. A Motherpack consisting of soap, a baby shawl, cotton wool, a cup, a spoon, a bathing towel, sanitary towels, and a basin was given to each mother who delivered at a health facility. These two interventions (TBA reorientation and the provision of Motherpacks) were implemented at different points in time for each health centre and the community it serves.

## Intervention

The maternal and child health project, *Afya njema kwa mama na mtoto* (Swahili phrase translated as ‘good health to the mother and child’), aimed to improve maternal and child health outcomes in a county that experiences a relatively low facility delivery coverage of 39% [5]. The project ran from January 2013 to December 2016, in Narok County, Kenya (the data used in this study covers the first 35 months, i.e., from January 2013 to December 2015). It was funded by the European Union and Christian Aid to the tune of €1,548,082. Christian Aid, in collaboration with the county government of Narok, managed the implementation of the project via three partners: the Narok Integrated Development Programme, Trans Mara Rural Development Programme, and Community Health Partners. The project included two main strands, a supply side health systems support and strengthening component, and a demand side component that sought to work with the community to remove key barriers to skilled delivery. The supply aspect included collaboration with the country government to construct two maternity facilities, renovate five health facilities in remote parts of the county, equip needy facilities with delivery equipment and improve referral services through the purchase of two ambulances. The demand side was strengthened, firstly, by training TBAs to encourage women to make use of health facilities at childbirth and to accompany women in labour to health facilities^3^; and secondly, by providing Motherpacks to mothers who delivered at a health facility^4^. TBA reorientation and Motherpacks incentives were new interventions in this context; implemented at different times for each of the 28 health facilities between January 2013 and December 2015. The data used in this paper covers the first 35 months of the project cycle.

Of relevance to our project was the June 2013 announcement of free maternity services in all public health facilities throughout Kenya, after which no charges were levied to mothers for maternity services. This represented an effort by the government to boost the number of women giving birth in health facilities [6]. The Beyond Zero Campaign, which aims to ensure that ‘no woman dies giving a life’ [19], champions this cause on an ongoing basis. This represented another major incentive to the uptake of health facilities in our study area, and as such a major potential confounding variable. We therefore controlled for it in our model, as described in the analysis section.

## Methods^5^

The outcome variable is data on deliveries at 28 health facilities from the Narok County District Health Information System 2 (DHIS2) in which the interventions were implemented. The data for skilled deliveries used was collected at the maternity departments of each of the facilities using delivery registers between January 2013 and September 2015. It was assumed that health facility delivery data represents a reliable proxy for SBA delivery, and was therefore employed as a proxy for SBA delivery in the analysis. The intervention data consisted of the starting month for each of the two interventions at each of the 28 facilities and its catchment community unit. The data on dates interventions were implemented is obtained from reports by implementing agencies.

The analysis set out to estimate a dependency or inferred causality between the two interventions (provision of a Motherpack [variable: Motherpack], and reorientation of TBAs [variable: TbaEvent]), with the outcome (health facility delivery uptake as indicated by deliveries conducted by Skilled Birth Attendants at Health Facilities [variable: BirthsSBA]). Analytically, this dependency relationship is described as occurring between covariates (interventions) and variates (outcome). Thus, we have two covariates (Motherpack, TbaEvent), and one variate (BirthsSBA). The variables are all measured as count data, and we do not therefore believe the error distribution to be Normal. For this reason, we did not employ the oft-used ordinary least squared (OLS) regression. Instead, we used the generalized linear model (GLM) framework, which allows for a flexible specification of the error distribution [20]. GLMs have three components: the error distribution, the link function, and the linear predictor. For count data, the preferred GLM has a Poisson error distribution with the log link function, where the log is the natural logarithm function. Ordinarily, the Poisson distribution assumes equal mean and variance in the variate. This was found not to be the case for our data, as there was noticeable variance between the study facilities, and even within the study facilities, as indicated in Table 1, below.

**Table 1:** Mean and variance in health facility data

Facility	mean	variance
Aitong	7.11	12.39
Angata	25.97	323.06
Eawasongiro	3.58	11.91
Enaibora	2.11	5.40
Enosaen	17.14	124.42
Ilaser	3.19	9.93
Ilkerinloita	0.76	1.56
Ilomotiok	0.00	0.00
Kamaget	6.97	37.79
Kapsasian	7.64	45.89
Kimintet	2.00	1.60
Kojonga	3.67	5.66
Kurangurik	30.69	497.48
Mararianta	2.72	7.29
Naisoya	3.72	7.81
Njipship	2.94	8.34
Nkareta	1.53	2.08
Nturumenti	2.14	7.38
Oldekesi	2.56	7.63
Olmekenyu	10.50	117.74
Olokurto	12.94	72.29
Ololunga	42.42	159.39
Ongatanado	4.19	19.13
Romosha	1.94	3.48
Sakutiek	7.92	31.45
Sekenani	3.44	16.77
Sogoo	47.28	536.49
Talek	5.64	10.18

The Negative Binomial regression has become the traditional method to model overdispersed Poisson or count data [21]. Due to the discrepancy between mean and variance in our data, this was our natural choice. The introduction of free maternal healthcare by the Kenyan government was introduced into the model as a measure of months passed since its introduction [variable: months_since_free_Mcare].

For our implementation, we made use of R software [22], and used the glm.nb function from the R MASS library [23]. We fitted the following model:

- glm.nb (BirthsSBA ~ (months_since_TbaEvent +
- months_since_MPackEvent +
- months_since_free_Mcare)^2 +
- Facility*months_since_study_start),
- data = KenyaData, init.theta = 4.276890451, link = log)

The model tests the effects of the TBA reorientation events adjusted for time since intervention (variable: months_since_TbaEvent), the introduction of Motherpacks at health facilities, again adjusted for time since intervention (variable: months_since_MPackEvent), and the advent of free medical care provision by the Kenyan Government on 2013-06-23 (months_since_free_Mcare)^6^. We assigned a slope, with time, to each of these interventions; as well as a test for two-way interactions between each of these. We also applied an individual intercept and slope with time to each of the facilities to control for differences between facilities, as well as an overall effect of time (to control for an increase that may be caused by general population growth)^7^. Our model assumes that any intervention will have the same effect across all facilities, while controlling for interactions and the differences between the facilities. Table 2 shows the estimates and *p*-values of the parameters of interest, while the full list of outputs is given in the appendix.

**Table 2:** Estimates and *p*-values of interest

Coefficients:	Estimate	Std. Error z	value	Pr(>|z|
(Intercept)	1.4238	0.2299	6.2	6.00e-10***
months_since_TbaEvent	0.1547	0.059	2.6	0.009**
months_since_MPackEvent	0.2011	0.0458	4.4	1.00e-05***
months_since_free_Mcare	−0.0393	0.0281	−1.4	0.162
months_since_study_start	0.0513	0.024	2.1	0.033*
Interactions:				
months_since_TbaEvent:months_since_MPackEvent	0.0123	0.0045	2.7	0.006**
months_since_TbaEvent:months_since_free_Mcare	−0.0132	0.0042	−3.1	0.002**
months_since_MPackEvent:months_since_free_Mcare	−0.0059	0.0016	−3.6	3.00e-04***

Signif. codes: 0 ‘***’ 0.001 ‘**’ 0.01 ‘*’ 0.05 ‘.’ 0.1 ‘’ 1

## Results

From Table 2, we can see that both the TBA intervention and the Motherpacks intervention had significant positive effects on the number of births at a given health facility.

The interaction between the two interventions was also significantly positive, indicating that having both interventions results in a significantly better outcome than either one has on its own. The provision of free medical care failed to indicate a significant effect, when we controlled for the other interventions, thereby dismissing the possibility that the introduction of free medical care is alone related to the changes in the outcome. Interestingly, the interaction between the free medical care and the other two interventions is significant, but negative. We have interpreted this as the interventions providing an initial boost to the attendance, but that this boost wanes over time when free medical care is present. Consider the prediction equation for our model:

$$ \widehat{y}= \exp \left({\alpha}_0+{\beta}_1{x}_1+{\beta}_2{x}_2+{\beta}_3{x}_3+{\beta}_{12}{x}_1{x}_2+{\beta}_{23}{x}_2{x}_3+{\beta}_{13}{x}_1{x}_3+{\alpha}_{facility}+{\beta}_{facility}{x}_4\right) $$

See Table 3 for model variables and meanings, and Table 4 for estimates of significance levels, effect sizes and interactions from the whole range of variables in the model equation above.

**Table 3:** Model variables and meanings

Variable	Meaning
*x*_1_	months_since_TbaEvent
*x*_2_	months_since_MPackEvent
*x*_3_	months_since_free_Mcare
*x*_4_	months_since_study_start

**Table 4:** Significance levels, effect sizes and interactions

Coefficients:	Estimate	Std. Error	z value	Pr(>|z|)	
(Intercept)	1.42E + 00	2.30E-01	6.192	5.96E-10	***
months_since_TbaEvent	1.55E-01	5.90E-02	2.624	0.008682	**
months_since_MPackEvent	2.01E-01	4.58E-02	4.389	1.14E-05	***
months_since_free_Mcare	−3.93E-02	2.81E-02	−1.399	0.161872	
FacilityAngata	7.22E-01	2.80E-01	2.579	0.009902	**
FacilityEawasongiro	−9.40E-01	4.51E-01	−2.084	0.037125	*
FacilityEnaibora	−2.41E + 00	4.79E-01	−5.024	5.05E-07	***
FacilityEnosaen	7.81E-01	2.84E-01	2.747	0.006014	**
FacilityIlaser	−1.20E + 00	3.73E-01	−3.22	0.00128	**
FacilityIlkerinloita	−4.74E + 00	9.09E-01	−5.22	1.79E-07	***
FacilityIlomotiok	−3.36E + 01	1.42E + 06	0	0.999981	
FacilityKamaget	−9.49E-01	3.28E-01	−2.898	0.00375	**
FacilityKapsasian	−1.37E + 00	3.40E-01	−4.023	5.74E-05	***
FacilityKimintet	−1.20E + 00	3.69E-01	−3.253	0.001142	**
FacilityKojonga	−3.20E-01	3.33E-01	−0.962	0.336282	
FacilityKurangurik	2.02E-01	2.86E-01	0.705	0.480559	
FacilityMararianta	−2.16E + 00	4.21E-01	−5.132	2.86E-07	***
FacilityNaisoya	−1.00E + 00	3.84E-01	−2.618	0.008851	**
FacilityNjipship	−1.54E + 00	3.72E-01	−4.147	3.37E-05	***
FacilityNkareta	−1.59E + 00	4.08E-01	−3.894	9.86E-05	***
FacilityNturumenti	−1.95E + 00	4.13E-01	−4.727	2.27E-06	***
FacilityOldekesi	−2.15E + 00	4.18E-01	−5.141	2.74E-07	***
FacilityOlmekenyu	−1.50E + 00	3.41E-01	−4.399	1.09E-05	***
FacilityOlokurto	2.28E-01	3.03E-01	0.752	0.451866	
FacilityOlolunga	1.81E + 00	2.79E-01	6.479	9.26E-11	***
FacilityOngatanado	−2.44E + 00	4.26E-01	−5.726	1.03E-08	***
FacilityRomosha	−1.09E + 00	3.70E-01	−2.937	0.003319	**
FacilitySakutiek	−1.06E-01	3.18E-01	−0.332	0.739813	
FacilitySekenani	−2.79E + 00	4.56E-01	−6.106	1.02E-09	***
FacilitySogoo	1.62E + 00	2.71E-01	5.976	2.29E-09	***
FacilityTalek	−1.19E-01	3.05E-01	−0.389	0.69711	
months_since_study_start	5.13E-02	2.41E-02	2.135	0.032776	*
months_since_TbaEvent:months_since_MPackEvent	1.23E-02	4.49E-03	2.734	0.006256	**
months_since_TbaEvent:months_since_free_Mcare	−1.33E-02	4.22E-03	−3.138	0.001699	**
months_since_MPackEvent:months_since_free_Mcare	−5.93E-03	1.65E-03	−3.6	0.000319	***
FacilityAngata:months_since_study_start	3.53E-02	1.40E-02	2.522	0.011662	*
FacilityEawasongiro:months_since_study_start	−8.77E-02	4.29E-02	−2.047	0.040659	*
FacilityEnaibora:months_since_study_start	4.45E-02	2.31E-02	1.928	0.053906	.
FacilityEnosaen:months_since_study_start	1.37E-02	1.50E-02	0.912	0.361873	
FacilityIlaser:months_since_study_start	8.77E-03	1.98E-02	0.444	0.657226	
FacilityIlkerinloita:months_since_study_start	1.04E-01	3.44E-02	3.03	0.002443	**
FacilityIlomotiok:months_since_study_start	6.67E-02	7.09E + 04	0	0.999999	
FacilityKamaget:months_since_study_start	4.49E-02	1.53E-02	2.942	0.003261	**
FacilityKapsasian:months_since_study_start	7.24E-02	1.60E-02	4.536	5.73E-06	***
FacilityKimintet:months_since_study_start	−2.04E-04	1.75E-02	−0.012	0.990708	
FacilityKojonga:months_since_study_start	−2.85E-02	1.89E-02	−1.507	0.131897	
FacilityKurangurik:months_since_study_start	6.57E-02	1.42E-02	4.641	3.46E-06	***
FacilityMararianta:months_since_study_start	4.93E-02	1.84E-02	2.68	0.007363	**
FacilityNaisoya:months_since_study_start	−2.87E-02	2.58E-02	−1.113	0.265541	
FacilityNjipship:months_since_study_start	3.81E-02	1.70E-02	2.234	0.025499	*
FacilityNkareta:months_since_study_start	−2.87E-03	1.92E-02	−0.15	0.881119	
FacilityNturumenti:months_since_study_start	3.61E-02	1.83E-02	1.968	0.049067	*
FacilityOldekesi:months_since_study_start	5.29E-02	1.82E-02	2.901	0.003719	**
FacilityOlmekenyu:months_since_study_start	7.97E-02	1.52E-02	5.246	1.56E-07	***
FacilityOlokurto:months_since_study_start	1.24E-02	1.70E-02	0.73	0.465201	
FacilityOlolunga:months_since_study_start	4.16E-03	1.55E-02	0.269	0.787684	
FacilityOngatanado:months_since_study_start	7.62E-02	1.84E-02	4.134	3.56E-05	***
FacilityRomosha:months_since_study_start	−9.42E-03	1.92E-02	−0.491	0.623103	
FacilitySakutiek:months_since_study_start	−2.00E-03	1.82E-02	−0.11	0.912752	
FacilitySekenani:months_since_study_start	8.70E-02	1.88E-02	4.618	3.87E-06	***
FacilitySogoo:months_since_study_start	1.18E-02	1.31E-02	0.904	0.365875	
FacilityTalek:months_since_study_start	−3.17E-03	1.49E-02	−0.213	0.831417	

Signif. codes: 0 ‘***’ 0.001 ‘**’ 0.01 ‘*’ 0.05 ‘.’ 0.1 ‘’ 1

Here, ${\upbeta}_{\mathrm{j}}$ is the parameter associated with variable i and β_ij_ is the parameter for the interaction between variables i and j, as given in the results table.

**Fig. 1:**
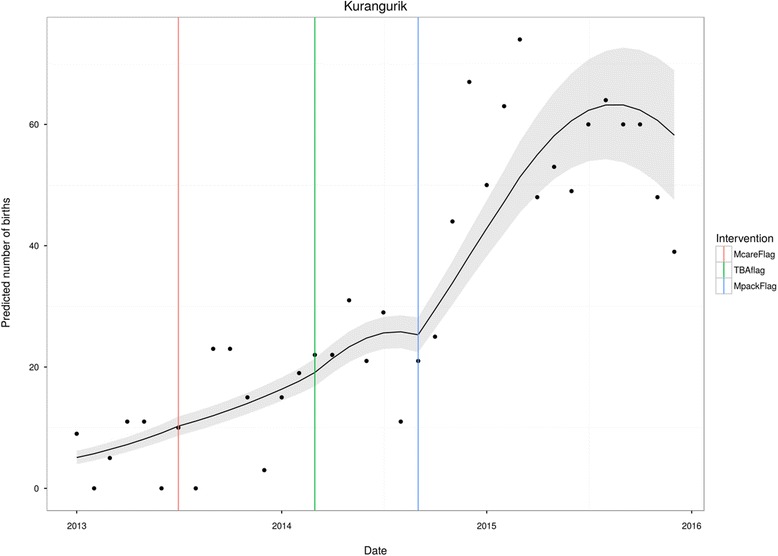
Kurangurik

**Fig. 2:**
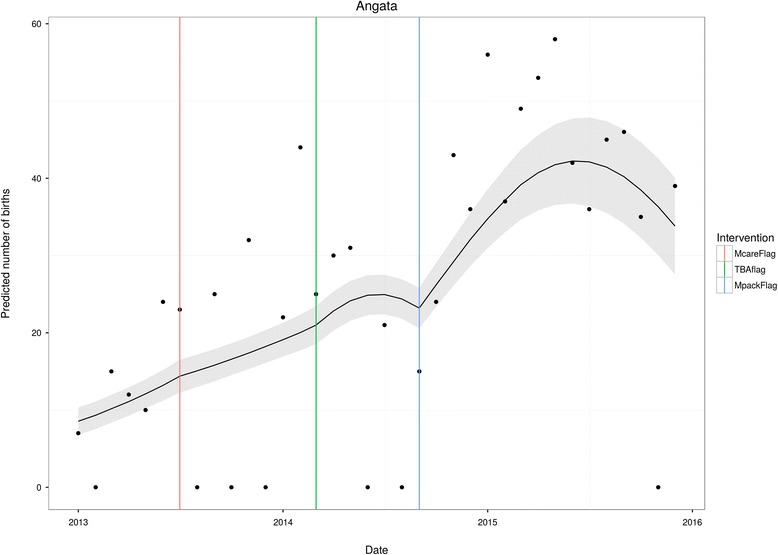
Angata

**Fig. 3:**
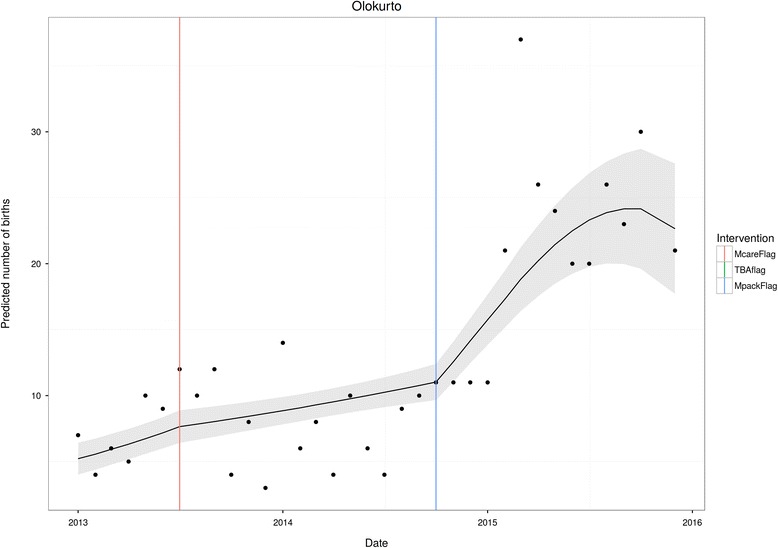
Olokurto

**Fig. 4:**
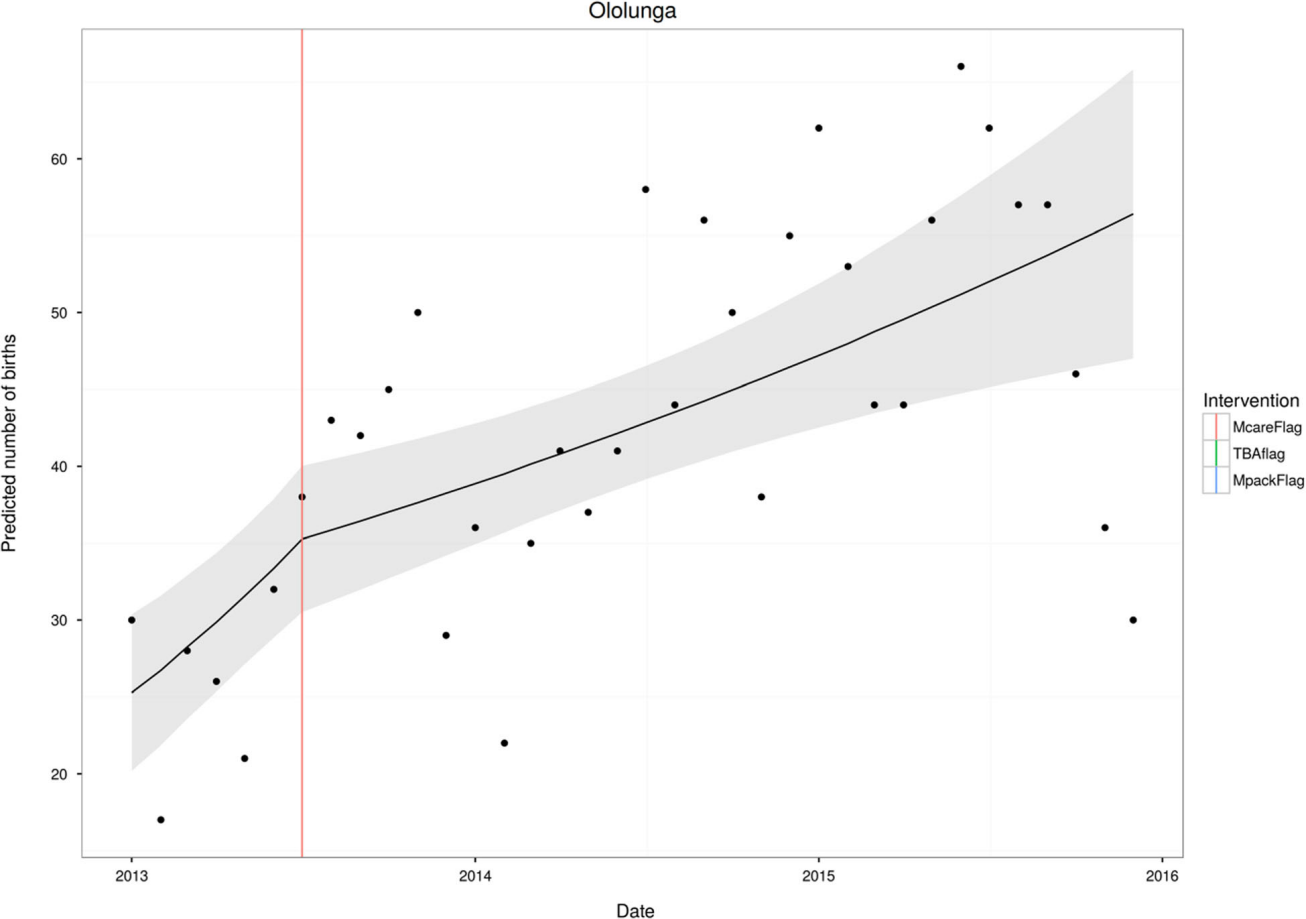
Ololunga

Figures 1, 2, 3 and 4 are plots of the model predictions at selected locations. The vertical lines indicate where the interventions started: McareFlag flags the initiation of free health care by the government; TBAFlag flags the initiation of the TBA reorientation for the health facility in question; and MpackFlag flags the initiation of the Motherpacks intervention for the health facility in question. Figures 1 and 2 present health facilities that were exposed to all interventions, i.e., free healthcare, TBA intervention, and Motherpacks intervention. In both, it is evident that initiation of free health care on its own has little effect on the slope of childbirths at the facility. The addition of the TBA intervention has a moderate effect on raising childbirths, while the Motherpacks intervention raises the number of childbirths most noticeably. Both TBA and Motherpacks interventions illustrate a marked attenuation over time.

Not all facilities were exposed to both TBA and Motherpacks interventions. In the case of Olokurto, TBA reorientation did not take place. Here, the marked impact of Motherpacks is evident (as is the lack of impact of free health care).

Data also included a small number of health facilities that were exposed to free health care only. Ololunga presents such a case. Here, the non-impact of free health care is evident.

## Discussion

### Summary of results

During the 35 months of the project cycle, a total of 9095 health facility deliveries took place. Within this period, free health care was introduced, a total of 408 TBAs were reached with reorientation activities, and 2181 Motherpacks were distributed to women delivering at health facilities. The introduction of free health care was found to have no significant effect on the outcome (*p* = 0.162). The reorientation of TBAs was significant (*p* = 0.009), as was the provision of Motherpacks (*p* = 0.0001). The number of months that passed since the start of the intervention was also found to be significant (*p* = 0.033). The introduction of Motherpacks had the greatest effect on the outcome (0.2), followed by TBA intervention (0.15). Months since study start had a much lower effect (0.05). Comparisons of effect sizes should be done with caution, as a model with many interactions, such as this one, results in complex and unclear interpretation of the effect sizes.

### Results in context

These empirical results show that both Motherpacks and TBA reorientation can play a critical role in influencing where mothers give birth in rural poor communities with a high proportion of deliveries happening outside health facilities. This appears to provide some evidence that far more positive results are obtained when TBAs are co-opted into health systems and taken on as allies in the struggle against childbirth related mortality, than when they are vilified and criminalised. TBAs appear to exert noticeable influence in their communities, and reorientation and incentivising strategies channel this pre-existing access and influence toward formal health systems, thereby increasing the uptake of facility delivery. These results are consistent with findings from elsewhere, that have recommended integration of TBAs into formal health systems [16]. The success of the Motherpacks intervention on health facility delivery indicates that useful and practical baby care commodities can serve as an effective non-financial incentive toward health facility uptake in similar rural contexts. This effect is very likely enhanced by resource poverty of rural communities in the Narok county, as such commodities may allow households to subsidize their expenses once the child is born, thereby increasing the opportunity cost of delivering at home for both the mother and care givers. Such realignment of household incentives appears to shift preferences and increase the uptake of facility delivery services where these are available and affordable alongside the Motherpacks.

The findings of this study suggest that, in Narok county at least, free health care on its own does not have a noticeable impact on whether mothers visit health facilities. Intuition suggests that this claim should be made with caution, as there is plenty of other evidence that would suggest otherwise. One possibility, however, is that because of free health care, women prefer to visit larger health facilities (and not the level one community health facilities that were the source of our data). Another possible explanation might be that the provision of free health care is not sufficient – on its own – to overcome the barriers to facility based delivery that lead women to undergo non-health facility childbirth in the first place. These barriers have been found to include – in the Kenyan context - perceptions of the skills of doctors versus TBA’s; the perceived likelihood of embarrassment or fear of being shamed; the knowledge of pregnancy risk factors and those affecting safe delivery; and the location of any previous deliveries. Also, higher facility childbirth was found in male headed households; and amongst those with higher education. Outside of Kenya, yet other barriers have been identified, and their relevance to the topic may be usefully investigated. These include transportation issues, fear of maltreatment, and others. Further investigation might usefully identify the nature of these barriers in order to enhance our study’s finding that additional interventions are required to shift influence and opportunity costs and draw women toward SBA health facility deliveries. Demand side interventions such as providing commodities to mothers after delivery, and training, reorienting, and financially incentivising TBAs while safeguarding their status and recognition in the community, can shift behaviours toward health facility deliveries over home deliveries.

### Policy implications

Working with TBAs, by reorienting and incentivising them toward cooperation, providing an enabling environment for their integration with health facilities, and providing mothers with basic childcare commodities, can significantly increase the use of skilled services in rural communities that still have a strong bias toward TBAs. Public investment into demand side financing and sustaining these interventions in LMICs can result in greater uptake of health facility delivery and contribute to improvements in maternal and new born health outcomes. In Kenya, and in other contexts where health facilities are supposed to be reimbursed for each delivery conducted, for example, using part of the reimbursement to invest in these innovative initiatives in contexts with high home deliveries by TBAs may improve return on investment to the local health facility as the numbers of deliveries at the respective facilities increase. Policy makers may usefully consider the extent to which these interventions will be sustainable should governments withdraw from their basic responsibilities of financing public health services as a result of recessions for example. Within the broader strategy to improve maternal and newborn health indicators, female education, women empowerment, provision of functional emergency obstetric care services near to the people, and removal of barriers such as user fees, among others should continue to play a critical and sustainable role complemented by innovative context specific efforts such as TBA reorientation and mother packs.

### Strengths and limitations

The selection of community units to implement the TBA reorientation and Motherpacks activities was focused in areas with greatest need and to facilities serving some remote areas of the Narok County in the spirit of the project’s objectives. This may mean that this evidence is not generally applicable across other dissimilar contexts.

In addition, there is some evidence of an overall secular trend toward increased facility delivery not just based on population growth, but based on shifting norms toward facility delivery [24, 25]. Further investigation into the impact of such a secular trend will go towards understanding the extent of this effect; if large enough, this may indicate that our adjusting for population growth was not on its own sufficient.

It would have been useful to conduct a comparison of interventions. For this, however, it would have been necessary to design data collection differently, so that at 25% of the facilities, no intervention would take place; at a further 25% of the facilities, one of the interventions would take place; at another 25% of the facilities, the other intervention would take place; and at 25% of the facilities, both interventions would take place. The interventions should still be randomly assigned within the 25-25-25-25 split in order to minimise the risk of bias in the assignments. Practical restrictions imposed by the project precluded such a design. As it is, the data allows interpretation only so far as effect size consideration is concerned.

### Future research recommendations

Given the long duration necessary in changing social norms, it will be important to research the effect of TBA reorientation on uptake of services in the long term, in order to monitor shifts in sociocultural norms that could be sustained without the financial incentives, for example. A qualitative study will be useful in exploring how TBA reorientation and Motherpacks increase uptake of facility delivery. Also, some understanding of the attenuation of effects will be useful, in order to modify interventions toward being more sustainable. We recommend an economic evaluation to access the true cost of implementing these interventions and assess the cost-effectiveness of adopting such a strategy in resource poor settings to inform safe motherhood programmes and policy makers’ choices in such an investment in Kenya or elsewhere. In addition, a better understanding of barriers to facility delivery would be useful.

## Conclusion

TBA reorientation and motherpacks incentives had significant positive effects on the number of births at a given health facility within a rural community with high home deliveries assisted by TBAs. Having both interventions resulted in a significantly better outcome than either one has on its own.

Collaborating with TBAs and offering basic commodities important to mothers and babies (Motherpacks) immediately after delivery at health facilities, can play a critical role in influencing where mothers give birth in rural poor communities that maintain a strong bias for TBA assisted home delivery. Because of their influence, far more positive results can be obtained in such contexts when TBAs are co-opted into health systems and taken on as allies and resource persons in the struggle against childbirth related mortality, than when they are vilified and criminalised.

Public investment into demand side incentives such as motherpacks and sustaining these interventions in LMICs can result in greater uptake of health facility delivery improving perinatal maternal and child health outcomes. Evidence on the cost-benefit of such investments is needed.

## Additional file

Additional file 1: Kitui_MCHKenya CodeBook and Kitui_MCHKenyaData. (ZIP 23 kb)

## Abbreviations

DHIS: District Health Information System
LMICs: Low and middle income countries
MCH: Maternal and child health
SBAs: Skilled birth attendants
TBAs: Traditional birth attendants

## Footnotes

1 It is important to note that there are notable variations in health facility delivery between different counties and communities in Kenya. Coverage ranges from as low as 18.3% in Wajir County to as high as 93.4% in Kiambu County

2 USD5 as of August 2016

3 The incentives for TBAs were instilled through improved education and a Ksh500 cash incentive for each referral

4 A Motherpack consisted of soap, baby shawl, cotton wool, cup, spoon, bathing towel, sanitary towels and a basin. Each Motherpack cost an average of Ksh750

5 The dataset and codebook supporting the conclusions of this article are included within the article (and its additional files).

6 The advent of free medical care by the Kenyan government was considered a major potential confounding variable, and was therefore controlled for by inclusion in the model.

7 “months_since_TbaEvent” indicates the time passed since the TBA reorientation, “months_since_MPackEvent” indicates the time passed since Motherpacks were introduced at Health Facilities, “months_since_free_Mcare” indicates the time passed since the introduction of free healthcare by the Kenyan Government, and “months_since_study_start” indicates the months since the start of the study, or the natural population growth
